# Forsythiasides-Rich Extract From Forsythiae Fructus Inhibits Mast Cell Degranulation by Enhancing Mitochondrial Ca^2+^ Uptake

**DOI:** 10.3389/fphar.2021.696729

**Published:** 2021-06-14

**Authors:** Ruijuan Qi, Yuan Kang, Ximeng Li, Xiaoyu Zhang, Yixin Han, Runlan Cai, Yuan Gao, Yun Qi

**Affiliations:** Institute of Medicinal Plant Development, Chinese Academy of Medical Sciences & Peking Union Medical College, Beijing, China

**Keywords:** forsythiae fructus, mast cell degranulation, IgE, Mrgpr, cytosolic Ca2+, mitochondrial Ca2+

## Abstract

Mast cells (MCs) activated via IgE/FcεRI or MAS-related G protein coupled receptor (Mrgpr)-mediated pathway can release granules that play prominent roles in hypersensitivity reactions. Forsythiae Fructus, a well-known traditional Chinese medicine, has been clinically used for allergic diseases. Although previous studies indicated that Forsythiae Fructus extract inhibited compound 48/80-induced histamine release from MCs, its effect on IgE-dependent MC degranulation and possible underlying mechanisms remain to be explored. Herein, we prepared the forsythiasides-rich extract (FRE) and investigated its action on MC degranulation and explored its underlying mechanism. Our data showed that FRE could dampen IgE/FcεRI- and Mrgpr-mediated MC degranulation *in vitro* and *in vivo*. Mechanism study indicated that FRE decreased cytosolic Ca^2+^ (Ca^2+^_[c]_) level rapidly and reversibly. Moreover, FRE decreased Ca^2+^_[c]_ of MCs independent of plasma membrane Ca^2+^-ATPase (PMCA), sarco/endoplasmic Ca^2+^-ATPase (SERCA) and Na^+^/Ca^2+^ exchanger (NCX). While, along with Ca^2+^_[c]_ decrease, the increase of mitochondrial Ca^2+^ (Ca^2+^_[m]_) occurred simultaneously in FRE-treated RBL-2H3 cells. In the isolated mitochondria, FRE also promoted the subcellular organelle to uptake more extramitochondrial Ca^2+^. In conclusion, by increasing Ca^2+^_[m]_ uptake, FRE decreases Ca^2+^_[c]_ level to suppress MC degranulation. Our findings may provide theoretical support for the clinical application of Forsythiae Fructus on allergy and other MC-involved diseases.

## Introduction

Mast cells (MCs), distributing at the host-environment interfaces, are a class of tissue-resident innate immune cells that can respond to various immunogenic stimuli in the first place. Generally, MCs can be activated via either IgE-dependent or IgE-independent pathways. In IgE-dependent pathway, MC activation can be initiated by crosslinking the IgE-FcεRI complexes with multivalent antigens, thereby resulting in MC degranulation. In IgE-independent pathway, MCs also possess unique responsiveness to the basic secretagogues [e.g., compound 48/80 (C48/80) and substance P], which can directly induce MC degranulation via activating the Mas-related G-protein coupled receptor (Mrgpr) (MrgprX2 for human MCs and MrgprB2 for mouse) (McNeil et al., 2015).

Although crosslinking of IgE-FcεRI complexes with antigens and activation of Mrgpr by C48/80 can induce different intracellular signal cascades, they ultimately reach the same outcome—elevation of cytosolic Ca^2+^ (Ca^2+^_[c]_) and subsequent MC degranulation (Chen et al., 2017). Further, the extracellular granules play prominent roles in hypersensitivity reactions (Wernersson and Pejler, 2014). Hence, preventing MC degranulation is regarded as an attractive therapeutic strategy in treating allergic diseases.

Forsythiae Fructus, the dried fruit of *Forsythia suspensa* (Thunb.) Vahl (Oleaceae), is a well-known traditional Chinese medicine (known as lianqiao) that has been thought of having heat-clearing and detoxifying effects on diseases like epidemic pyrexia, carbuncle, scrofula and erysipelas (Chen et al., 2015a), which might be attributed to its antibacterial/viral, anti-inflammatory, and antioxidant activities (Dong et al., 2017). Although Forsythiae Fructus extract blocked histamine release from MCs induced by C48/80 (Kim et al., 2003; Sung et al., 2016), its effect on IgE/FcεRI-mediated MC degranulation was not investigated. Given the clinical application of Forsythiae Fructus for the treatment of allergic diseases (Chien et al., 2013; Chen et al., 2015a), together with the fact that Shuang-Huang-Lian Injection containing Forsythiae Fructus markedly prevented IgE/FcεRI-mediated MC degranulation (Gao et al., 2017), it is very likely that Forsythiae Fructus can also dampen IgE/FcεRI-mediated MC degranulation.

Based on previous screening results of the active components in Forsythiae Fructus, we prepared the forsythiasides-rich extract (FRE) and demonstrated its inhibitory effect on MC degranulation and the underlying mechanism. Our findings not only enrich the existing understanding of the ethnomedicinal application of Forsythiae Fructus, but also exhibit FRE’s potential as an herbal preparation.

## Materials and Methods

### Reagents

C48/80, 4-Methylumbelliferyl N-acetyl-β-D-glucosaminide, bovine serum albumin (BSA), Pluronic F-127 and thapsigargin were purchased from Sigma-Aldrich (St Louis, MO, United States). Fluo-3 AM Ester, Calcium Green-5N and Cal-630 AM Ester were from Biotium (San Francisco, CA, United States), Invitrogen (Carlsbad, CA, United States) and MKbio (Shanghai, China), respectively. Plasmid pcDNA3-4mtD3cpv was from Beijing Zoman Biotechnology Co. Ltd. (Beijing, China). The transfection reagent Entranster^TM^-H4000 was from Engreen Biosystem (Beijing, China). Dulbecco’s modified Eagle’s medium (DMEM), fetal bovine serum (FBS), and trypsin were purchased from Gibco BRL (Grand Island, NY). Nycodenz was from Axis-shield (Scotland, United Kingdom). Forsythiaside A (CAS^#^79916-77-1), isoforsythiaside (CAS^#^1357910-26-9) and phillyrin (CAS^#^487-41-2) were from Baoji Herbest Bio-Tech Co. Ltd. (Baoji, Shaanxi, China, purity >98%). All the other reagents were of analytical grade.

### Preparation of Shrimp Protein (SP) and Anti-SP Serum

SP from *Penaeus japonicus* and the anti-SP serum were prepared as previously described (Gao et al., 2019).

### Preparation of FRE From Forsythiae Fructus

We first collected both unripe Forsythiae Fructus (UFF) and ripe Forsythiae Fructus (RFF) from different producing areas, and prepared the crude aqueous extracts for comparing their effects on β-hexosaminidase release. The obtained results showed that both effect and yield of UFF were better than that of RFF. To avoid disturbance of batch-to-batch variation, we chose UFF from Sangjiahe Zhendong Wild Medicinal Tending Base of Forsythiae Fructus (Shanxi, China) to prepare adequate FRE. Thus, UFF used in the present study was all from the same batch. Forsythiae Fructus was validated by Professor Yun Qi in Institute of Medicinal Plant Development (IMPLAD) of Chinese Academy of Medical Sciences (CAMS), China. A voucher specimen (IMPLAD-XL327) was deposited in the Herbarium of IMPLAD. Dried raw materials were shattered and refluxed in distilled water (1: 10, w/v) for 2 h. The obtained solution was precipitated with ethanol-water (85:15, v/v) at 4°C overnight (>20 h). The supernatant was concentrated and passed through the AB-8 macroporous resin column, followed by gradient ethanol elution 10% elution was discarded and 60% elution was collected and lyophilized (Shi et al., 2011). The final sample (FRE) represented a 9.68% yield of raw material dry weight and was stored at -20°C. It was dissolved in aqueous buffer (*in vitro* experiments) or saline (*in vivo* experiments) before used, and the concentrations and doses were chosen according to the preliminary experiments.

### HPLC Analysis

A Shimadzu LC-15C HPLC system (Shimadzu, Japan) equipped with LC solution software, a UV spectrophotometer detector (Shimadzu, Japan) and a Syncronis C18 column (4.6 mm × 250 mm, 5 μm; ThermoFisher Scientific, Massachusetts, United States) were used for HPLC analysis. And the sample (10 μl) was injected using an autosampler. The mobile phases were (A) 0.4% acetic acid and (B) acetonitrile with a gradient elution as listed in Table 1. The flow rate was 1 ml/min and the detection wavelength was 235 nm.

**TABLE 1:** Mobile phase condition of chromatographic separation.

(min)	A (0.4% acetic acid) (%)	B (acetonitrile) (%)
0	80	20
40	73	27
60	60	40
70	40	60
75	80	20

### Animals

All animal care and experimental procedures were complied with the National Institutes of Health Guide for Care and Use of Laboratory Animals and approved by the Institutional Animal Care and Use Committee of the IMPLAD of CAMS [SYXK (Beijing) 2017-0020]. BALB/c mice (male, 20–22 g) were purchased from Beijing Vital River Laboratory Animal Services (Beijing, China) and housed under specific pathogen-free conditions with a 12 h light/dark cycle with free access to standard diet and water. And the anesthetic and other necessary measures were used in order to reduce the animal suffering in studies.

### Cells

Rat basophilic leukemia cell line (RBL-2H3) was purchased from the cell bank of Chinese Academy of Sciences (Shanghai, China). Human LAD2 cell line (from Michael D. Gershon, MD, Columbia University, United States) was a gift from Prof. Renshan Sun (the Third Military Medical University, Chongqing, China). And mouse peritoneal mast cells (MPMCs) were isolated from the BALB/c mice.

### Cell Cytotoxicity Assay

LDH assay was chosen to predict the early cell damage of FRE (Cavallo et al., 2012; Gao et al., 2014). Cells (1 × 10^5^ cells/well) were cultured in a 96-well plate and treated with FRE (0–1 mg/ml) at 37°C for 2 h. The supernatant (70 μl) was incubated with 20 μl of lithium lactate solution (36 mg/ml, in 10 mM Tris buffer, pH 8.5) and 20 μl of INT solution (2 mg/ml in PBS, pH 7.2). The reaction was initiated by adding 20 μl of a mixture of NAD^+^ and diaphorase (3 mg/ml NAD^+^, 53.9 U/ml diaphorase, 0.03% BSA, and 1.2% sucrose in PBS, pH 7.2) and incubated at 25°C for 20 min. The absorbance was measured at 492 nm.

### β-Hexosaminidase Release Assay

The β-hexosaminidase release assay was performed following previously described (Gao et al., 2017). For the measurement of C48/80-induced β-hexosaminidase release, LAD2 cells or MPMCs were pretreated with FRE (200–600 μg/ml) at 37°C for 30 min followed by adding C48/80 (10 μg/ml) for a further 1.5 h incubation. Thirty microliters of supernatant were collected and mixed with 50 μl of substrate solution (0.57 mg/ml 4-Methylumbelliferyl N-acetyl-β-D-glucosaminide in 0.133 M sodium citrate buffer, pH 4.3) in a 96-well black flat bottom plate at 25°C for 2 h and the reaction was terminated by adding stop buffer (50 mM glycine and 5 mM EDTA·Na_2_, pH 10.5). The fluorescence intensity was read at λ _ex_ 355 nm/λ _em_ 460 nm by a fluorescence microplate analyzer (Thermo Scientific Fluoroskan Ascent FL, United States).

For IgE/FcεRI-mediated β-hexosaminidase release, 1% anti-SP serum sensitized-RBL-2H3 cells were pretreated with FRE for 30 min at 37°C and then co-incubated with SP (40 ng/ml) for another 1.5 h. The supernatant was collected for the β-hexosaminidase detection.

### Measurement of Ca^2+^_[c]_ Level

Ca^2+^_[c]_ level was measured using the Ca^2+^-reactive fluorescent probe Fluo-3 AM as previously described (Gao et al., 2021). For determining IgE/FcεRI-elevated Ca^2+^_[c]_ level, RBL-2H3 cells were sensitized by 1% anti-SP serum overnight and loaded with Fluo-3 AM (4 μM) at 30°C in dark for 30 min. Probenecid was added to block Fluo-3 AM leakage. After removing the extracellular dye, the stained cells were treated with FRE at 37°C for 30 min and then challenged by 40 ng/ml of SP. Ca^2+^_[c]_ level was immediately determined. For the measurement of C48/80-caused Ca^2+^_[c]_ increase, LAD2 cells were loaded with Fluo-3 AM as above described and treated with FRE at 37°C for 30 min. C48/80 (10 μg/ml) was added and the Ca^2+^_[c]_ level was immediately monitored.

### Measurement of Plasma Membrane Ca^2+^-ATPase (PMCA) and Sarco/Endoplasmic Ca^2+^-ATPase (SERCA) Activities

To measure whether the effect of FRE on Ca^2+^_[c]_ was due to activating PMCA or SERCA, the experiments were performed by using the alkaline pH (9.0) medium or thapsigargin (5 μM) to suppress the PMCA or SERCA activity, respectively (Gover et al., 2007; Peng and Guo, 2007). Briefly, RBL-2H3 cells loaded with Fluo-3 AM were resuspended in alkaline pH (9.0) medium or thapsigargin (5 μM) solution in the presence of FRE. Ca^2+^_[c]_ levels were immediately monitored.

### Measurement of Na^+^/Ca^2+^ Exchanger (NCX) Activity

The NCX activity assay was carried out as previously described with slight modifications (Heise et al., 2011). Ca^2+^_[c]_ level was monitored upon removal of external Na^+^ in the presence of external Ca^2+^, switching NCX into a reverse mode (Ca^2+^ entry mode). Briefly, RBL-2H3 cells loaded with Fluo-3 AM were resuspended in Na^+^ solution (95 mM NaCl, 2 mM CaCl_2_, 2 mM MgCl_2_, 10 mM HEPES, 40 mM KCl and 10 mM glucose, pH 7.4) or Na^0^ solution (95 mM NaCl was substituted by equivalent N-Methyl-d-glucamine). The cells of the two groups were then treated with FRE and the Ca^2+^_[c]_ levels were immediately determined.

### Fluorescent Image of Mitochondrial Calcium (Ca^2+^_[m]_) and Ca^2+^_[c]_

To simultaneously determine Ca^2+^_[c]_ and Ca^2+^_[m]_ levels in RBL-2H3 cells, the Ca^2+^-reactive fluorescent probe Cal-630 AM and the mitochondrial-targeted Ca^2+^-sensitive plasmid pcDNA-4mtD3cpv were used. Firstly, RBL-2H3 cells were transiently transfected with pcDNA-4mtD3cpv using Entranster^TM^-H4000 (Engreen Biosystem, China) according to the manufacture instruction. Forty-eight hours later, the cells were loaded with Cal-630 AM (4 μM) in HEPES buffer containing 0.04% (w/v) Pluronic F-127 and incubated at 37°C in dark for 90 min and further incubated at room temperature for another 30 min. At the end of incubation, probenecid (4 mM) was added to block Cal-630 AM leakage. After removing the extracellular dye, cells were treated with FRE or the equal volume of vehicle. The fluorescent image was immediately taken by a laser confocal fluorescence microscopy (Nikon A1) using a 60 × oil objective (The fluorescence intensity of Cal-630 AM was recorded at λ _ex_ 600 nm/λ _em_ 640 nm, and the fluorescence intensity of the mitochondrial-targeted biosensor was recorded at λ _ex_ 435 nm/λ _em_ 535 nm).

### Measurement of Ca^2+^_[m]_ Uptake of Isolated Mouse Liver Mitochondria

Ca^2+^_[m]_ uptake of isolated mouse liver mitochondria was also measured using Calcium Green-5N according to previously described (Baughman et al., 2011; Schmitt et al., 2015). Briefly, 100 µg isolated mitochondria were resuspended in KCl buffer (containing 0.5 µM Calcium Green-5N, 2.5 mM glutamate and 2.5 mM malate) in the presence of FRE. Fluorescence was monitored immediately after 50 µM CaCl_2_ was added.

### Drug Administration *In Vivo*

Appropriate route of drug administration is crucial for *in vivo* experiment. Whether oral gavage or intraperitoneal injection is both irreplaceable and frequently-used. Oral route is a well-accepted choice for herb extracts because it is more relevant to the ethnomedicinal application. By comparison, intraperitoneal injection is obviously closer to *in vitro* experiment because drug can directly contact with the effector cells without the influence of gastrointestinal tract. In this study, given that the used *in vivo* models is emergent and severe, we chose intraperitoneal injection instead of oral route.

### C48/80-Induced Hypothermia in Mice

The C48/80-induced hypothermia model was established as previously described with slight modifications (Gao et al., 2021). BALB/c mice were intraperitoneally injected (*i.p.*) with C48/80 (4 mg/kg), while the mice in the negative control group were received an equal volume of saline. Five minutes later, the mice were intraperitoneally injected with FRE or an equal volume of saline (for the negative control group and C48/80 alone group). Thirty minutes later, the rectal temperature was monitored by a thermal probe (Beijing Hongou Chengyun Instrument and Equipment Co., LTD., China).

### Passive Systemic Anaphylaxis (PSA)

Mouse PSA model was established as previously described (Gao et al., 2021). BALB/c mice were intravenously sensitized (*i.v.*) with anti-SP serum (30 μl/mouse). Twenty-four hours later, mice were pretreated (*i.p.*) with FRE or equal volume of saline (for the negative control group and SP alone group) for 30 min and then challenged by SP (200 μg/mouse, *i.v.*) except the negative control group. Twenty minutes after SP injection, the rectal temperature was measured.

### Statistical Analysis

The results were expressed as the mean ± SD. One-way analysis of variance (ANOVA) was used when more than two groups were compared using SPSS 20.0 software. The Tukey *post-hoc* analysis was followed for between-group comparison. Statistical analysis between two groups was performed by Students’ *t*-test. Differences at *p* < 0.05 were considered statistically significant.

## Results

### HPLC Analysis of FRE

**FIGURE 1:**
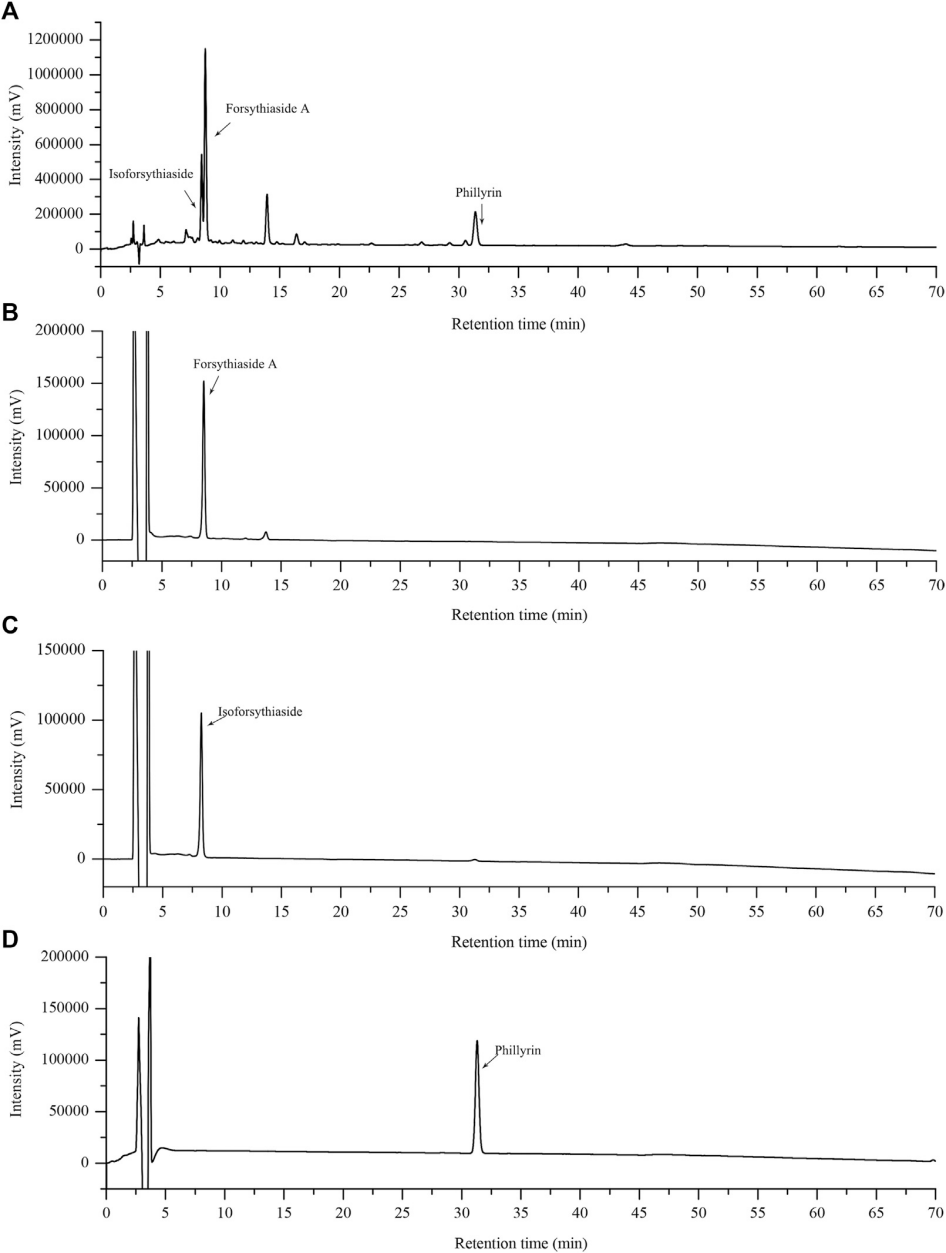
HPLC chromatograms of FRE and its characteristic components. The fingerprint of FRE **(A)** and the chromatograms of three marker components, forsythiaside A **(B)**, isoforsythiaside **(C)**, and phillyrin **(D)**, respectively.

The representative standard fingerprint of FRE was shown in Figure 1A. Three peaks were identified by matching retention time to the respective reference compounds, representing forsythiaside A, isoforsythiaside, and phillyrin. Their retention times were 9.187, 8.805 and 31.129 min (Figures 1B–D), and the contents in FRE were 27.47 ± 1.04%, 9.46 ± 0.12%, and 5.05 ± 0.29%, respectively.

### Inhibitory Effect of FRE on MC Degranulation *In Vitro*

**FIGURE 2:**
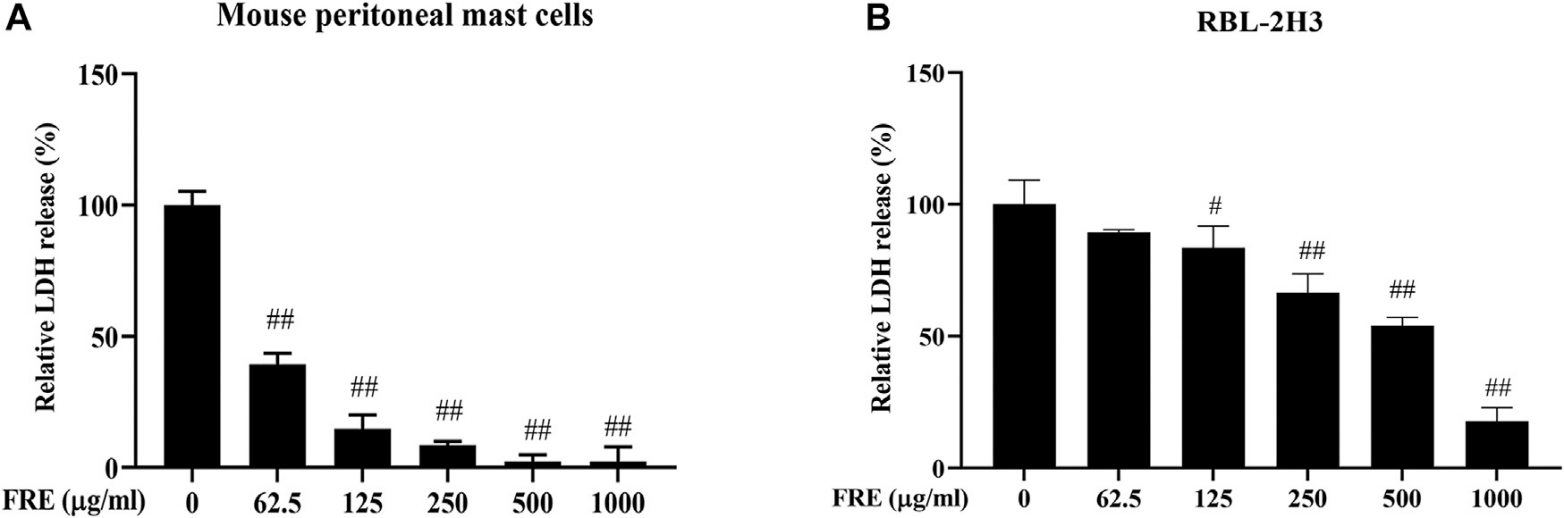
FRE did not impair cell viability of MPMCs **(A)** and RBL-2H3 cells **(B)** (*n* = 3). The cells were pretreated with FRE for 2 h and the extracellular LDH was determined. ^#^ *p* < 0.05, ^##^ *p* < 0.01 *vs.* control.

We first evaluated the effect of FRE on cell viability using extracellular LDH assay. After being incubated with FRE (200–600 µg/ml) for 2 h, LAD2 cells released undetectable LDH in the culture medium (data not shown). In RBL-2H3 cells and MPMCs, FRE did not increase, or rather, significantly decreased LDH leakage (Figures 2A,B). These findings demonstrated that FRE did not impair cell viability at the indicated concentrations.

**FIGURE 3:**
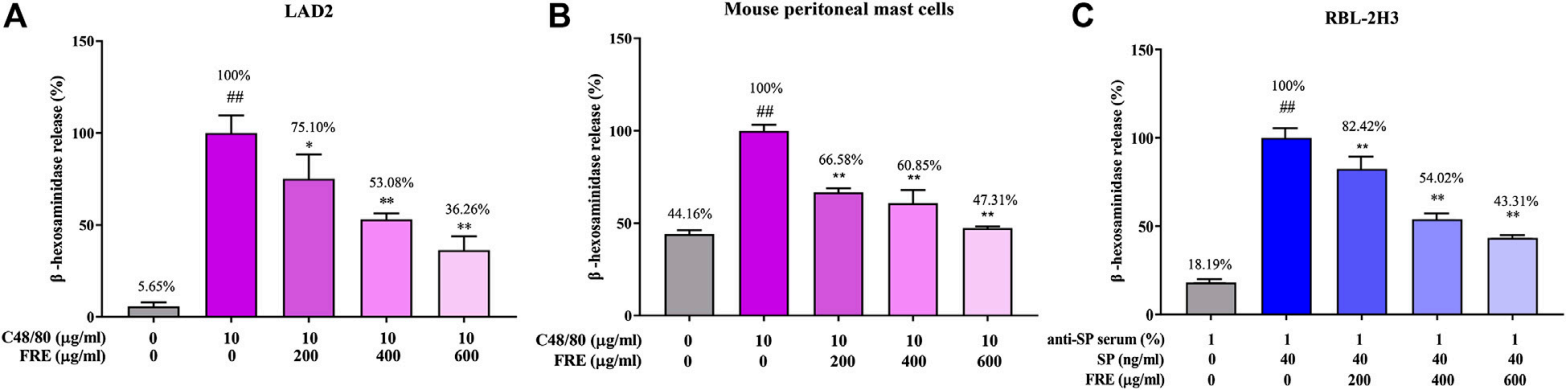
FRE inhibits both Mrgpr- and IgE/FcεRI-mediated MC degranulation *in vitro* (*n* = 3). **(A,B)** Effect of FRE on Mrgpr-mediated MC degranulation *in vitro*. LAD2 **(A)** and MPMCs **(B)** were pretreated with FRE for 30 min and then exposed to C48/80 (10 μg/ml) for another 1.5 h. Supernatants were collected for the β-hexosaminidase release assay. ^##^ *p* < 0.01 *vs.* control and *p < 0.05, **p < 0.01 *vs.* C48/80 alone group. **(C)** Effect of FRE on IgE/FcεRI-mediated MC degranulation *in vitro*. The sensitized RBL-2H3 cells were pretreated with FRE for 30 min and then challenged with SP. The β-hexosaminidase release assay was conducted 1.5 h after SP stimulation. ^##^ *p* < 0.01 *vs*. negative control (the anti-SP serum sensitization alone group) and **p < 0.01 *vs*. model group (the both anti-SP serum sensitization and SP challenge group).

Next, the *in vitro* effect of FRE on MC degranulation was investigated by detecting β-hexosaminidase release in the supernatants of LAD2, MPMCs and RBL-2H3 cells, respectively. Based on our previous observations, LAD2 and MPMCs are more suitable for the Mrgpr-mediated MC degranulation. Whereas, RBL-2H3 cells are more suitable for IgE/FcεRI-mediated MC degranulation (Zhang et al., 2020). Therefore, we detected C48/80-induced β-hexosaminidase release in LAD2 cells and MPMCs. As shown in Figures 3A,B, C48/80 (10 µg/ml) caused a significant release of β-hexosaminidase in these two cells (*p <* 0.01), while FRE could concentration-dependently suppressed the β-hexosaminidase release with IC_50_ value of 441.5 and 525 µg/ml, respectively.

Next, we assessed IgE/FcεRI-mediated MC degranulation in RBL-2H3 cells. The data showed that FRE reduced IgE/FcεRI-mediated β-hexosaminidase release with an IC_50_ value of 540.4 µg/ml (Figure 3C). Overall, these results indicated that FRE could dampen both Mrgpr- and IgE/FcεRI-mediated MC degranulation *in vitro*.

### Inhibitory Effect of FRE on MC Degranulation *In Vivo*

**FIGURE 4:**
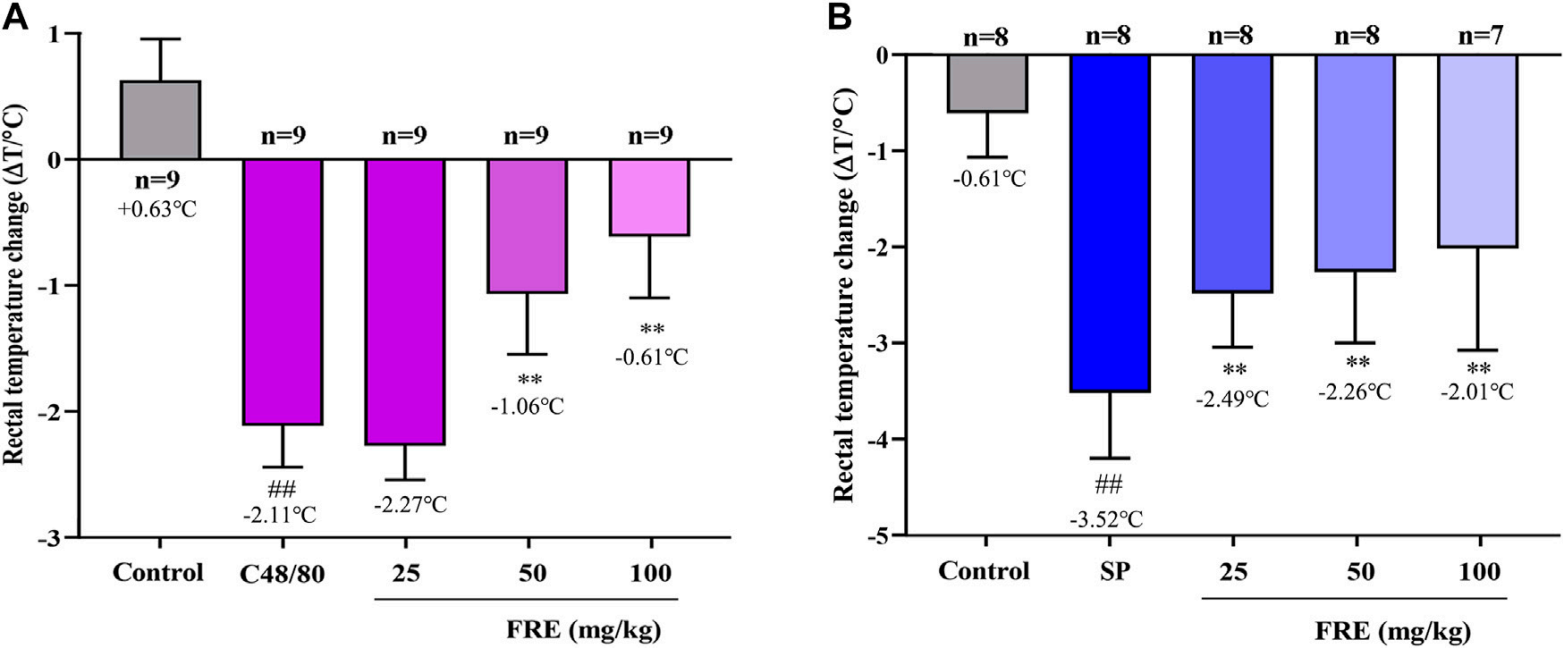
FRE suppresses the Mrgpr- and IgE/FcεRI-mediated MC degranulation *in vivo.* **(A)** Effect of FRE on the rectal temperature of C48/80-treated mice. Mice were *i.p.* injected C48/80 (4 mg/kg) 5 min before FRE administration (*i.p.*). Thirty minutes after C48/80 treatment, the rectal temperature was measured. ^##^ *p* < 0.01 *vs.* control and **p < 0.01 *vs.* C48/80 alone group. **(B)** Effect of FRE on the rectal temperature of PSA mice. The sensitized mice were pretreated with FRE (*i.p.*) for 30 min and then *i.v.* challenged with SP (200 μg/mouse). The rectal temperature was monitored 20 min later. ^##^ *p* < 0.01 *vs.* control and **p < 0.01 *vs.* SP alone group.

Since FRE significantly inhibited MC degranulation *in vitro*, we next explored its effect *in vivo*. As shown in Figure 4A, C48/80 led to a decrease of rectal temperature (ΔT = 2.11 ± 0.33°C) compared with the initial rectal temperature, while FRE (50 and 100 mg/kg) could significantly prevent the rectal temperature drop (*p* < 0.01). In IgE-mediated *in vivo* model, injection of SP markedly lowered the rectal temperature (ΔT = 3.52 ± 0.68°C), while FRE could significantly counter the hypothermia (Figure 4B), indicating that FRE could inhibit MC degranulation *in vivo*.

### FRE Reduces Ca^2+^_[c]_ Level of LAD2 and RBL-2H3 Cells

**FIGURE 5:**
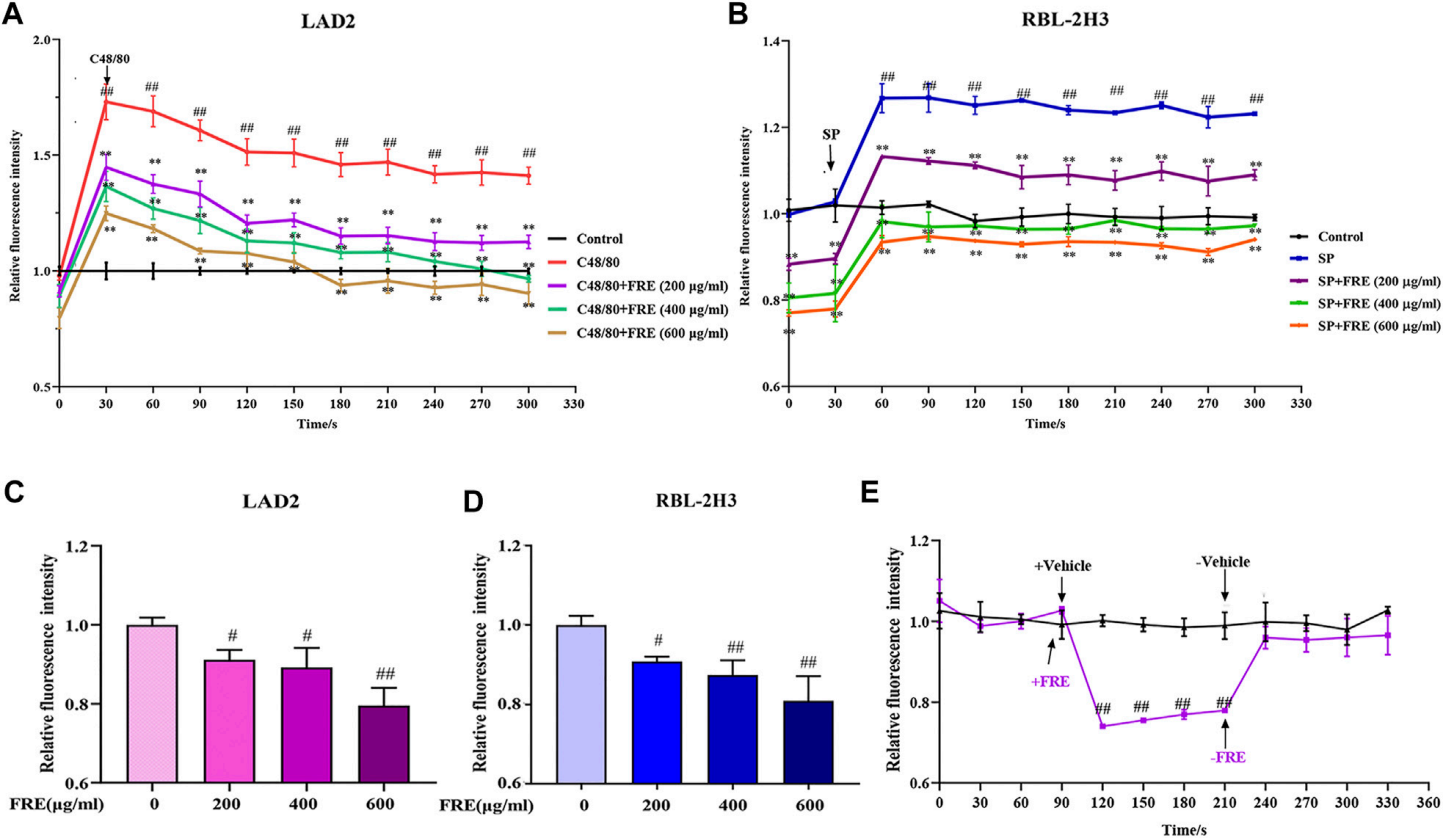
FRE concentration-dependently reduces the Ca^2+^_[c]_ level (*n = 3*). **(A,B)** FRE reduces C48/80-elicted Ca^2+^_[c]_ rise in LAD2 cells **(A)** and IgE/FcεRI-mediated Ca^2+^ increase in sensitized RBL-2H3 cells **(B)**. Cells were loaded with Fluo-3 AM (4 μM) at 30°C for 30 min and then treated with or without FRE for 30 min and exposed to C48/80 (10 μg/ml) or SP (40 ng/ml). The fluorescence intensity (λ e_x_ 485 nm/λ _em_ 538 nm) was detected every 30 s. ^##^ *p* < 0.01 *vs.* control, **p < 0.01 *vs.* SP or C48/80 alone. **(C,D)** FRE reduces Ca^2+^_[c]_ level in the resting LAD2 **(C)** and RBL-2H3 cells **(D)**. ^#^ *p* < 0.05 and ^##^ *p* < 0.01 *vs*. control. **(E)** FRE reduces the Ca^2+^_[c]_ level in a rapid and reversible manner in RBL-2H3 cells. ^##^ *p* < 0.01 *vs.* control.

Given the consistently inhibitory effects of FRE on MC degranulation both *in vitro* and *in vivo*, we wondered whether FRE suppressed Ca^2+^_[c]_ elevation, which was a crucial event that triggered the degranulation of MC. Expectedly, either C48/80 or SP challenge could markedly elevate Ca^2+^_[c]_ level, while FRE could concentration-dependently buffer Ca^2+^_[c]_ elevation (Figures 5A,B). Notably, FRE decreased Ca^2+^_[c]_ before C48/80 or SP challenge, suggesting that the effect of FRE on Ca^2+^_[c]_ was independent of these two pathways. Thus, we next investigated the effects of FRE on Ca^2+^_[c]_ in resting LAD2 and RBL-2H3 cells. As expected, FRE indeed rapidly decreased Ca^2+^_[c]_ of these two cells (Figures 5C,D). Moreover, Ca^2+^_[c]_ level of RBL-2H3 cells could be restored to the homeostasis once FRE was withdrawn (Figure 5E), indicating that FRE reduced the Ca^2+^_[c]_ level in a rapid and reversible way.

### FRE Decreases the Ca^2+^_[c]_ Level Independent of PMCA, SERCA and NCX

**FIGURE 6:**
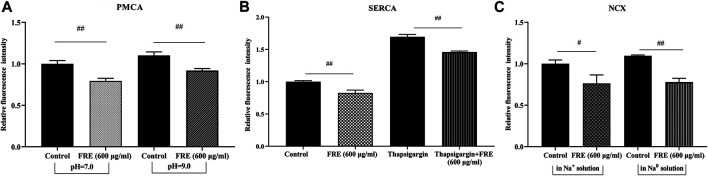
FRE decreases Ca^2+^_[c]_ independent of PMCA, SERCA and NCX in RBL-2H3 cells (*n = 3*). **(A)** Effect of PMCA on FRE-induced Ca^2+^_[c]_ reduction. The PMCA activity was determined by shifting the extracellular pH to 9.0. ^##^ *p* < 0.01. **(B)** Effect of SERCA on FRE-induced Ca^2+^_[c]_ reduction. Thapsigargin (5 μM) was used to inhibit SERCA activity. ^##^ *p* < 0.01. **(C)** Effect of NCX on FRE-induced Ca^2+^_[c]_ reduction. The NCX activity was detected in a Na^0^ solution containing 40 mM KCl. ^#^ *p* < 0.05, ^##^ *p* < 0.01.

The rapid and reversible effect of FRE on Ca^2+^_[c]_ in the resting cells strongly suggested a non-genomic mechanism. Thus, we further sought its underlying mechanisms. Typically, Ca^2+^_[c]_ reduction can be achieved by pumping Ca^2+^ out of cells via NCX and/or PMCA, or transporting Ca^2+^ into the endoplasmic reticulum (ER) through SERCA (Gover et al., 2007; Peng and Guo, 2007). We investigated their roles in FRE-induced Ca^2+^_[c]_ decrease one by one in RBL-2H3 cells. As shown in Figures 6A,B, the effect of FRE on Ca^2+^_[c]_ reduction was not affected in the condition of suppressing PMCA or SERCA activity by adjusting extracellular pH to 9 or adding thapsigargin, respectively. Moreover, when reversing NCX activity by replacing the extracellular Na^+^ with N-Methyl-d-glucamine, FRE was still able to decrease Ca^2+^_[c]_ (Figure 6C). These results indicated FRE decreased Ca^2+^_[c]_ independent of PMCA, SERCA and NCX.

### FRE Decreases Ca^2+^_[c]_ Level via Increasing Ca^2+^_[m]_ Uptake

**FIGURE 7:**
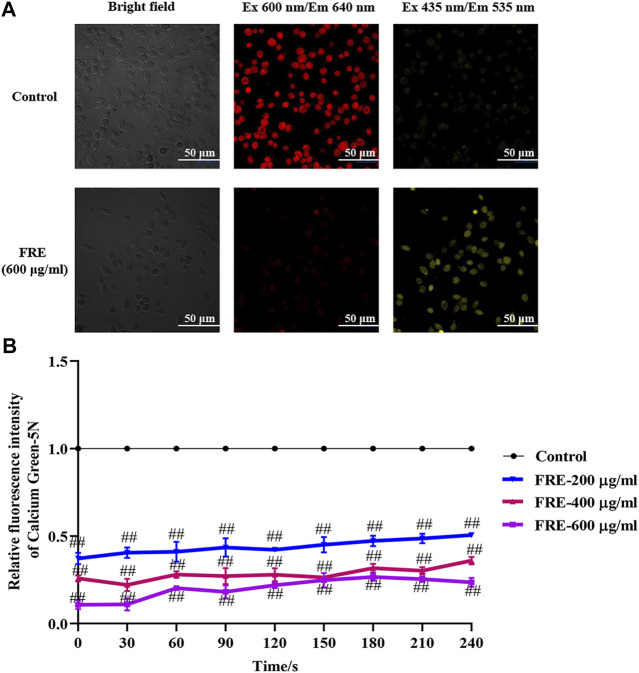
FRE increases Ca^2+^_[m]_ uptake (*n* = *3*). **(A)** Representative images of FRE induced Ca^2+^_[c]_ decrease and Ca^2+^_[m]_ increase in RBL-2H3 cells. Cells were transfected with pcDNA-4mtD3cpv. Forty-eight hours later, cells were then loaded with Cal-630 AM. After removing the dye, cells were treated with FRE (600 μg/ml) or the equal volume of vehicle (control). The fluorescent images were immediately taken by a laser confocal fluorescence microscopy (Nikon A1) using a 60 × oil objective (Cal-630 AM, λ _ex_ 600 nm/λ _em_ 640 nm; pcDNA-4mtD3cpv, λ _ex_ 435 nm/λ _em_ 535 nm). Scale bar represents 50 μm. **(B)** FRE increases Ca^2+^_[m]_ uptake in the isolated mouse liver mitochondria. ^##^ *p* < 0.01 *vs.* control.

Besides the above three ways, mitochondria uptake of Ca^2+^ is also a classical way for Ca^2+^_[c]_ reduction. If FRE-induced Ca^2+^_[c]_ reduction was through enhancing Ca^2+^_[m]_ uptake, the phenomenon of Ca^2+^_[c]_ decrease along with Ca^2+^_[m]_ increase should be observed. Thus, we designed an experiment to simultaneously measure the Ca^2+^_[m]_ and Ca^2+^_[c]_. To visually assay Ca^2+^_[m]_, a mitochondria-selective Ca^2+^ fluorescent plasmid pcDNA-4mtD3cpv was used (Palmer et al., 2006). Given the spectral overlap of pcDNA-4mtD3cpv (λ _ex_ 435 nm/λ _em_ 535 nm) and Fluo-3 AM (λ _ex_ 485 nm/λ _em_ 538 nm), we deliberately chose an alternative Ca^2+^ fluorescent dye Cal-630 AM (λ _ex_ 600 nm/λ _em_ 640 nm) to avoid interfering with each other. As expected, we indeed observed an obvious decrease in red fluorescence (Ca^2+^_[c]_) concurrent with an obvious increase in yellow fluorescence (Ca^2+^_[m]_) in RBL-2H3 cells (Figure 7A), demonstrating that FRE decreased Ca^2+^_[c]_ through enhancing Ca^2+^_[m]_ uptake. To evaluate whether the effect of FRE on Ca^2+^_[m]_ was direct or indirect, we next determined its effect on isolated mouse liver mitochondria by a membrane-impermanent Ca^2+^ fluorescent probe Calcium Green-5N. When Ca^2+^_[m]_ uptake increases, the extra-mitochondrial Ca^2+^ decreases along with the fluorescence intensity decreasing. As shown in Figure 7B, FRE could markedly decrease the extra-mitochondrial Ca^2+^ in a concentration-dependent manner (*p* < 0.01), indicating that the intra-mitochondrial Ca^2+^ was increased. These findings revealed that FRE decreased Ca^2+^_[c]_ mainly through a direct action on mitochondria.

## Discussion

Upon extracellular stimulation, MCs can be activated via IgE/FcεRI or Mrgpr-mediated signaling and consequently release lots of preformed granules which contain many biologically active mediators (e.g., β-hexosaminidase and tryptases) (Wernersson and Pejler, 2014). However, these two pathways possess a distinct pattern of granule release. IgE/FcεRI-mediated pathway leads to more histamine release, whereas Mrgpr-mediated pathway leads to more tryptase B2 release. Therefore, they exhibit different pathological outcomes (Gaudenzio et al., 2016). Clinically, the antihistamine’s therapeutic strategy is only effective in IgE/FcεRI-associated itch (Meixiong et al., 2019). However, our data showed that FRE could dampen MC degranulation induced by not only IgE/FcεRI-mediated, but also Mrgpr-mediated pathway (Figure 3), while the latter was consistent with previous findings (Kim et al., 2003; Sung et al., 2016). Perhaps not coincidentally, the clinical application of Forsythiae Fructus includes but not limited to IgE/FcεRI-associated pruritus, such as eczema (Chen et al., 2015b), urticaria (Chien et al., 2013), senile skin pruritus (Yang et al., 2018), uremic pruritus (Dong et al., 2018) and atopic dermatitis (Bardana, 2004; Chen et al., 2015a), which may be attributed to its inhibitory effect on two types of MC degranulation.

Cross-linking of FcεRI initiates a complex cascade of intracellular tyrosine kinases, leads to Ca^2+^_[c]_ elevation, and eventually triggers MC degranulation (Wernersson and Pejler, 2014). Mrgpr-mediated pathway shares a similar mechanism of inducing Ca^2+^_[c]_ elevation with IgE/FcεRI pathway (Gaudenzio et al., 2016), nevertheless its intracellular signaling events are not as well understood. It was previously found that these two signaling pathways could elicit 5–6-fold increase of Ca^2+^_[c]_ before MC degranulation. Moreover, Ca^2+^ rise induced by IgE/FcεRI pathway is found to be higher and more sustained than that induced by Mrgpr-mediated pathway (Chen et al., 2017). In our study, C48/80-elicited rapid increase of Ca^2+^_[c]_ in LAD2 cells and SP-induced sustained Ca^2+^_[c]_ increase in sensitized RBL-2H3 cells were observed, while FRE was able to suppress Ca^2+^_[c]_ elevation in a rapid and reversible fashion (Figure 5).

Ca^2+^_[c]_ can be extruded out of cells via PMCA and/or NCX, or sequestered into ER and/or mitochondria (Ma and Beaven, 2009). After screening one by one, PMCA, NCX and SERCA were excluded (Figure 6). To our knowledge, mitochondria are able to accumulate large amounts of Ca^2+^ inside their matrix (10–20-fold more Ca^2+^ than the cytosolic compartment) during Ca^2+^_[c]_ increasing, thus markedly buffering the Ca^2+^_[c]_ rise (Giorgi et al., 2018). Our data showed that FRE could increase Ca^2+^_[m]_ concomitantly with Ca^2+^_[c]_ decrease in RBL-2H3 cells (Figure 7A), indicating that FRE reduced Ca^2+^_[c]_ via increasing Ca^2+^_[m]_. Moreover, in the isolated mitochondria, FRE still promoted Ca^2+^_[m]_ uptake (Figure 7B), showing a direct effect on mitochondria. As we know, the process of Ca^2+^ entry into the mitochondria matrix is mainly via the mitochondria Ca^2+^ uniporter (MCU) complex, a Ca^2+^-activated Ca^2+^ channel, and is driven by the electrochemical gradient that exists across the mitochondrial inner membrane (ΔΨm) (Giorgi et al., 2018). Therefore, we tried to further explore whether FRE-induced Ca^2+^_[m]_ increase was attributed to MCU. Unfortunately, because of the spectral interference from FRE itself, the application of ruthenium red, an MCU inhibitor, was unavailing in our experiment.

Ca^2+^_[m]_ is crucial for cellular ATP production and erobic respiration. Upon Ca^2+^_[m]_ rise, the Ca^2+^-sensitive dehydrogenases of Krebs cycle are activated, facilitating the oxidative phosphorylation and thus promoting the ATP synthesis (Bhosale et al., 2015). However, when excessive Ca^2+^ enters into mitochondria (Ca^2+^_[m]_ overload), it can cause overproduction of reactive oxygen species (ROS), a by-product of Krebs cycle, which in turn impairs the bioenergetic metabolism and thus eventually triggers cell apoptosis (Bhosale et al., 2015). Obviously, it is critical to maintain Ca^2+^_[m]_ homeostasis. Likewise, buffering Ca^2+^_[c]_ through Ca^2+^_[m]_ uptake to dampen MC degranulation also requires such a balance, which is to intake Ca^2+^ into mitochondria effectively without causing Ca^2+^_[m]_ overload. In the present study, FRE was effective and non-toxic in both *in vivo* and *in vitro* models, indicating that the effect of FRE on Ca^2+^_[m]_ increase was sustainable to a certain degree. Moreover, the non-toxicity of FRE under the effective concentrations might also be related to its reversible feature on reducing Ca^2+^_[c]_. In addition, the characteristic components in FRE, including forsythiaside A, isoforsythiaside, and phillyrin (Figures 1B–D), possess ROS scavenging activity (Qu et al., 2012; Wei et al., 2014; Gong et al., 2020). Indeed, we also found that FRE could potently scavenge intracellular ROS of MCs (data not shown).

**FIGURE 8:**
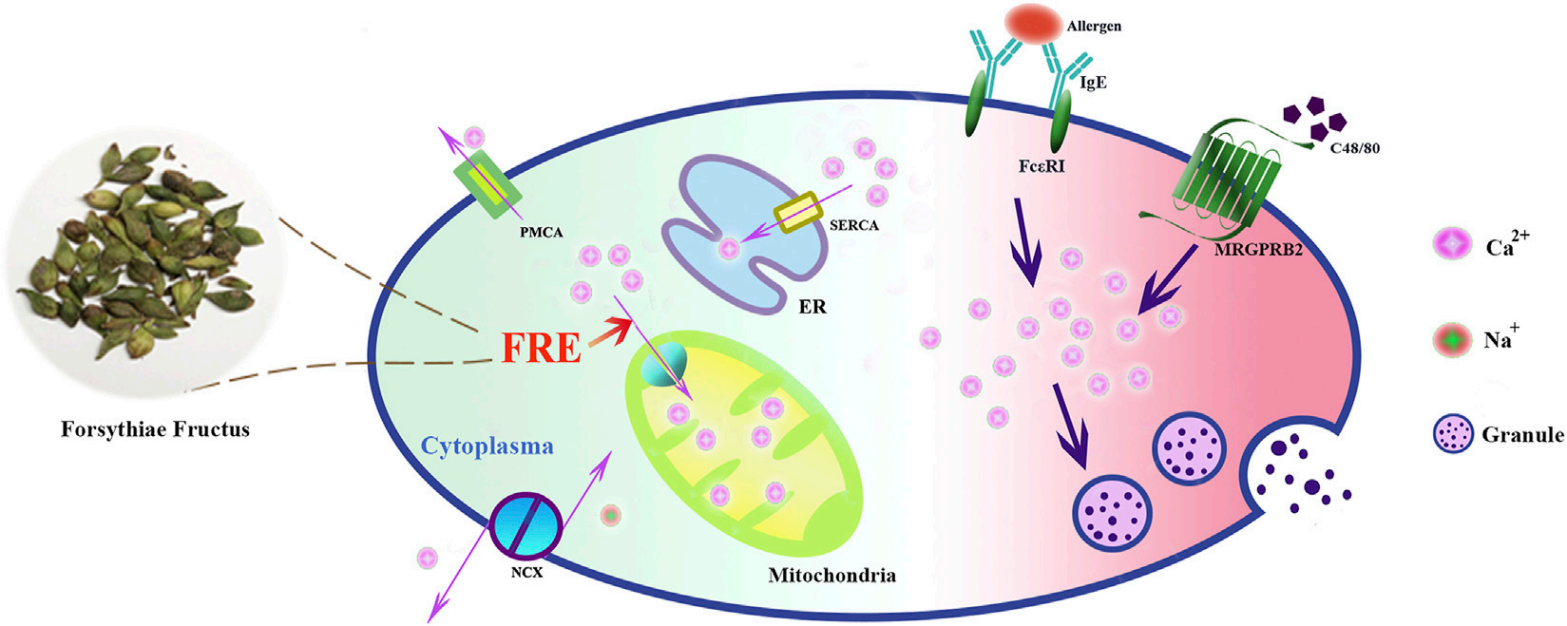
Schematic graph depicting how FRE dampened MC degranulation. FRE decreased Ca^2+^_[c]_ by enhancing Ca^2+^_[m]_ uptake, thereby suppressed IgE/FcεRI-mediated and Mrgpr-mediated MC degranulation.

In summary, our study reveals that FRE promotes mitochondria to uptake more Ca^2+^ to buffer the IgE/FcεRI- or Mrgpr-caused Ca^2+^_[c]_ rise, and consequently inhibits MC degranulation (Figure 8). These findings provide theoretical support for the clinical applications of Forsythiae Fructus in allergic diseases, and may also enlarge its clinical use scope in other MC-involved non-allergic disorders.

## Data Availability

The raw data supporting the conclusions of this article will be made available by the authors, without undue reservation.

## Abbreviations

C48/80, compound 48/80; Ca^2+^_[c]_, cytosolic Ca^2+^; Ca^2+^_[m]_, mitochondrial Ca^2+^; FRE, forsythiasides-rich extracts; MCs, mast cell; Mrgpr, the mas-related G-protein receptor; MPMCs, mouse peritoneal mast cells; RFF, ripe forsythiae fructus; SP, shrimp protein; UFF, unripe forsythiae fructus.
